# Comparing stereotactic ablative radiotherapy (SABR) versus re-trans-catheter arterial chemoembolization (re-TACE) for hepatocellular carcinoma patients who had incomplete response after initial TACE (TASABR): a randomized controlled trial

**DOI:** 10.1186/s12885-019-5461-3

**Published:** 2019-03-28

**Authors:** Liang-Cheng Chen, Wen-Yen Chiou, Hon-Yi Lin, Moon-Sing Lee, Yuan-Chen Lo, Li-Wen Huang, Chun-Ming Chang, Tsung-Hsing Hung, Chih-Wen Lin, Kuo-Chih Tseng, Dai-Wei Liu, Feng-Chun Hsu, Shih-Kai Hung

**Affiliations:** 1 Department of Radiation Oncology, Dalin Tzu Chi Hospital, Buddhist Tzu Chi Medical Foundation, No. 2, Min-Sheng Road, Dalin, Chia-Yi, Taiwan, Republic of China; 2 Department of Gastroenterology, Dalin Tzu Chi Hospital, Buddhist Tzu Chi Medical Foundation, Chia-Yi, Taiwan, Republic of China; 3 Department of General Surgery, Dalin Tzu Chi Hospital, Buddhist Tzu Chi Medical Foundation, Chia-Yi, Taiwan, Republic of China; 4 Department of Radiology, Dalin Tzu Chi Hospital, Buddhist Tzu Chi Medical Foundation, Chia-Yi, Taiwan, Republic of China; 5 Department of Radiation Oncology, Buddhist Tzu Chi General Hospital, Hualien, Taiwan, Republic of China; 6 School of Medicine, Buddhist Tzu Chi University, Hualien, Taiwan, Republic of China; 7 Institute of Molecular Biology, National Chung Cheng University, Min-Hsiung, Chia-Yi, Taiwan, Republic of China

**Keywords:** Hepatocellular carcinoma (HCC), Trans-arterial chemoembolization (TACE), Stereotactic ablative radiotherapy (SABR), Stereotactic body radiation therapy (SBRT)

## Abstract

**Background:**

Hepatocellular carcinoma (HCC) accounts for 75–85% of primary liver cancers and is prevalent in the Asia-Pacific region. Till now, trans-arterial chemoembolization (TACE) is still one of common modalities in managing unresectable intermediate-stage HCC. However, post-TACE residual viable HCC is not uncommon, resulting in unsatisfied overall survival after TACE alone. Recently, stereotactic ablative radiotherapy (SABR) has been suggested to manage HCC curatively. However, evidence from phase-III trials is largely lacking.

Hence, the present phase III randomized trial is designed to compare clinical outcomes between SABR and re-TACE for unresectable HCC patients who had incomplete response after initial TACE.

**Methods:**

The present study is an open-label, parallel, randomized controlled trial. A total of 120 patients will be included into two study groups, i.e., SABR and re-TACE, with a 1:1 allocation rate. A 3-year allocating period is planned. Patients with incomplete response after initial TACE will be enrolled and randomized. The primary endpoint is 1-year freedom-form-local-progression rate. Secondary endpoints are disease-progression-free survival, overall survival, local control, response rate, toxicity, and duration of response of the treated tumor.

**Discussion:**

SABR has been reported as an effective modality in managing intermediate-stage HCC patients, but evidence from phase-III randomized trials is largely lacking. As a result, conducting randomized trials to demarcate the role of SABR in these patients is warranted, especially in the Asia-Pacific region, where HBV- and HCV-related HCCs are prevalent.

**Trial registration:**

Before enrolling participants, the present study was registered prospectively on ClinicalTrials.gov (trial identifier, NCT02921139) on Sep. 29, 2016. This study is ongoing.

## Background

### Background and rationale

#### Hepatocellular carcinoma (HCC)

Liver cancers are sixth cause of cancer incidence and third cause of cancer death over the world – estimated 818,000 patient died in 2013 [1]. Of these, HCC accounts for 75–85% of primary liver cancers and is the third cause of cancer death in the Asia-Pacific region, where are also the endemic areas of chronic viral hepatitis (mainly hepatitis B and C) [2–4], leading to a combination of liver function impairment, such as liver cirrhosis, in HCC patients.

In addition to adverse effects of chronic viral hepatitis, most HCC patients were classified as intermediate to advanced stage at the time of diagnosis. As a result, though many treatment modalities can be chose for these patients [5, 6], 5-year survival rate is still poor, being less than 20% [7]. Note that the main failure pattern after tumor resection is intra-liver recurrence [8]. This observation still holds true for patients who undergo TACE or other local treatment modality alone.

#### Trans-arterial chemoembolization (TACE)

Although a Cochrane review failed to show significant survival benefits of TACE for unresectable HCC patients [9], TACE is still the most widely used locoregionally life-extending treatment for HCC patients with intermediate stage [6]. This recommendation is mainly based on six randomized prospective studies that demonstrate a survival benefit of TACE when compared with best supportive care or suboptimal therapies [10]. Note that most previously mentioned studies are investigated about ‘unresectable’ rather than “intermediate stage” HCC that classified by BCLC classification [11].

#### Stereotactic ablative radiotherapy (SABR)

When compared with conventional RT, SABR used a very precise way to delivering high dose of irradiation in a limited number of treatment fractions, demonstrating a large therapeutic benefit [12]. For maintaining a high precision of SABR, reliable immobilization, daily on-board image guidance, respiratory gating, and 360-degree Volumetric-Modulated Arc Therapy (VMAT) techniques are conducted clinically [13].

Recently, SABR, or alternatively named stereotactic body radiotherapy (SBRT), has been reported as a curative modality in managing early-stage HCC [14–21]. Moreover, the role of SABR is gradually defined in HCC patients with relatively large size (i.e., > 3 cm) [22], advanced [23], unresectable [24], and oligometastatic disease [25]. Remarkably, a role of SABR has been also reported in post-TACE HCC patients, in terms of adjuvant [26] or salvage setting [27, 28]. However, till now, level-III evidence is still largely lacking, rising an importance to conduct randomized trials for this issue [29–31].

Most studies showed that local control of inoperable HCC treated with SABR are about 72–89.8% at 1 year and 64% at 2 years, respectively [32, 33]. For selected patients, local control can be achieved as high as 99% at 1 and 2 years, respectively [34]. These outcomes of SABR were comparable with RFA, particularly for tumors > 2 cm [24, 35]. More notably, SABR showed a benefit of limited toxicities, even in elderly HCC patients [36].

### Rationale to conduct SABR for incomplete TACE

As mentioned above, TACE is effective for managing HCC patients with early to intermediate stage. However, TACE alone cannot achieve satisfactory complete tumor response rate, demonstrating median response and complete response rates after TACE was 38% (range 3–86%) and 0 (range 0–35%), respectively [37]. The unsatisfactory complete response suggested the concept of combining local therapy to improve local control.

In this regard, TACE combined with RFA has been reported to be useful in local tumor control [38, 39]. Recently, image-guided high-precise radiotherapy, i.e., SABR, has been reported to play a role in managing incomplete TACE [27]. However, evidence from randomized phase III studies is largely lacking.

Hence, the main reason to conduct SABR for unresectable HCC patients who had incomplete response after TACE is to increase treatment response and to prolong patient survival [24, 26, 28, 40–45]. In addition, post-TACE embolized materials were also useful to guide dose delivery of SABR, enhancing targeting precision.

### Objective and hypothesis

Taken together, conducting a randomized clinical trial to test the role of SABR in eradicating HCC – particularly for post-TACE residual tumors – is strongly encouraged [29–31]. As a result, the present phase-III trial intends to compare clinical outcomes between TACE plus SABR and TACE plus re-TACE for HCC patients who had post-TACE residual tumors.

#### Primary objective

The main goal of the present study is to assess the role of SABR in HCC patients who have incomplete response after initial TACE. Patients treated with re-TACE will be allocated into the active comparator group. Freedom form local progression (FFLP) is the primary end point.

#### Secondary objectives

Several secondary objectives are as follows: overall survival, tumor response rate, duration of tumor response, and side effects.

#### Hypothesis

We hypothesize that SABR is able to demonstrate better clinical outcomes than that of re-TACE for HCC patients who have incomplete response after their initial TACE, in terms of tumor response, local-progression-free and patient survival. In addition, limited treatment toxicities are expected to be observed in patients treated with SABR, as reported previously [45].

#### Trial design

The present study is a prospective, parallel, and open-label randomized controlled trial (RCT). An allocation ratio of 1:1 will be applied between the two study groups, i.e., SABR and re-TACE.

## Methods/design

### Ethic and consent

The present study will be conducted after a formal approval of Institute Review Board (Dalin Tzu Chi Hospital, Buddhist Tzu Chi Medication Foundation; approval number: A10502001). Important protocol modifications, e.g., changes to eligibility criteria or outcome analyses, will be re-submitted to IRB and implemented only after a re-approval of IRB.

Written informed consent will be obtained for each participant. Details of the trial process, including pros and cons of interventions, will be explained by both physician and study nurse. Whole-day contact information of investigators will be provided for all participants and their families. Obtained data will be kept in a security locker. The present protocol is reported according to recommendation of SPIRIT 2013 [46]. Final analyses and results will be submitted for publication on a peer-reviewed journal after completion of the study.

### Study setting

The present study will be conducted at a single regionally academic institute (i.e., Dalin Tzu Chi Hospital, Chia-Yi, Taiwan). Inter-institute cooperation for enrolling potential patients may be done under formal supervision of IRBs.

### Inclusion criteria

Several inclusion criteria will be as follows.

1. Patients who are diagnosed with HCC via one of the following methods:
  - radiographically typical enhancement pattern with arterial enhancement and portal or delayed washed out on triple-phase dynamic Computed Tomography (CT) or Magnetic Resonance Imaging (MRI), with or without an elevated value of alpha-fetoprotein (AFP); or,
  - histopathological confirmation of HCC.
2. HCC patients who are treated with initial TACE with incomplete response (i.e., partial response, stable disease, or disease progression).
3. Age ≥ 20 years old.
4. BCLC stage A-B and Child-Pugh score A-B.
5. Unresectable tumors, medically inoperable status, or refusal of surgery.
6. Clinically feasible for SABR or re-TACE.
7. SABR can be applied within 6 weeks of registration.
8. ECOG 0–1.
9. Life expectancy > 3 months.
10. Negative pregnancy.
11. No prior treatment, except for surgical resection and radiofrequency ablation (RFA).
12. Criteria of allowed laboratory data were as follows:
  - hemoglobin, ≥8.0 g/dl (may be post-transfusion if clinically indicated);
  - total bilirubin, ≤3.0 mg/dl;
  - AST (SGOT), ≤5-fold institutional upper limit of normal range;
  - ALT (SGPT), ≤5-fold institutional upper limit of normal range;
  - absolute neutrophil count, ≥1000/cumm;
  - platelet count, ≥20,000/cumm (may be post-transfusion if clinically indicated); and,
  - prothrombin time, international normalized ratio, ≥1.7.

### Exclusion criteria

1. Prior radiotherapy to the upper abdomen.
2. Prior other malignancy – unless disease free for at least 3 years.
3. Medical condition unsuitable such as cardiac ischemia or cerebrovascular accident within last 6 months.
4. Psychosocial condition unsuitable.
5. History of sorafenib therapy within 21 days prior.

### Interventions

**Fig. 1:**
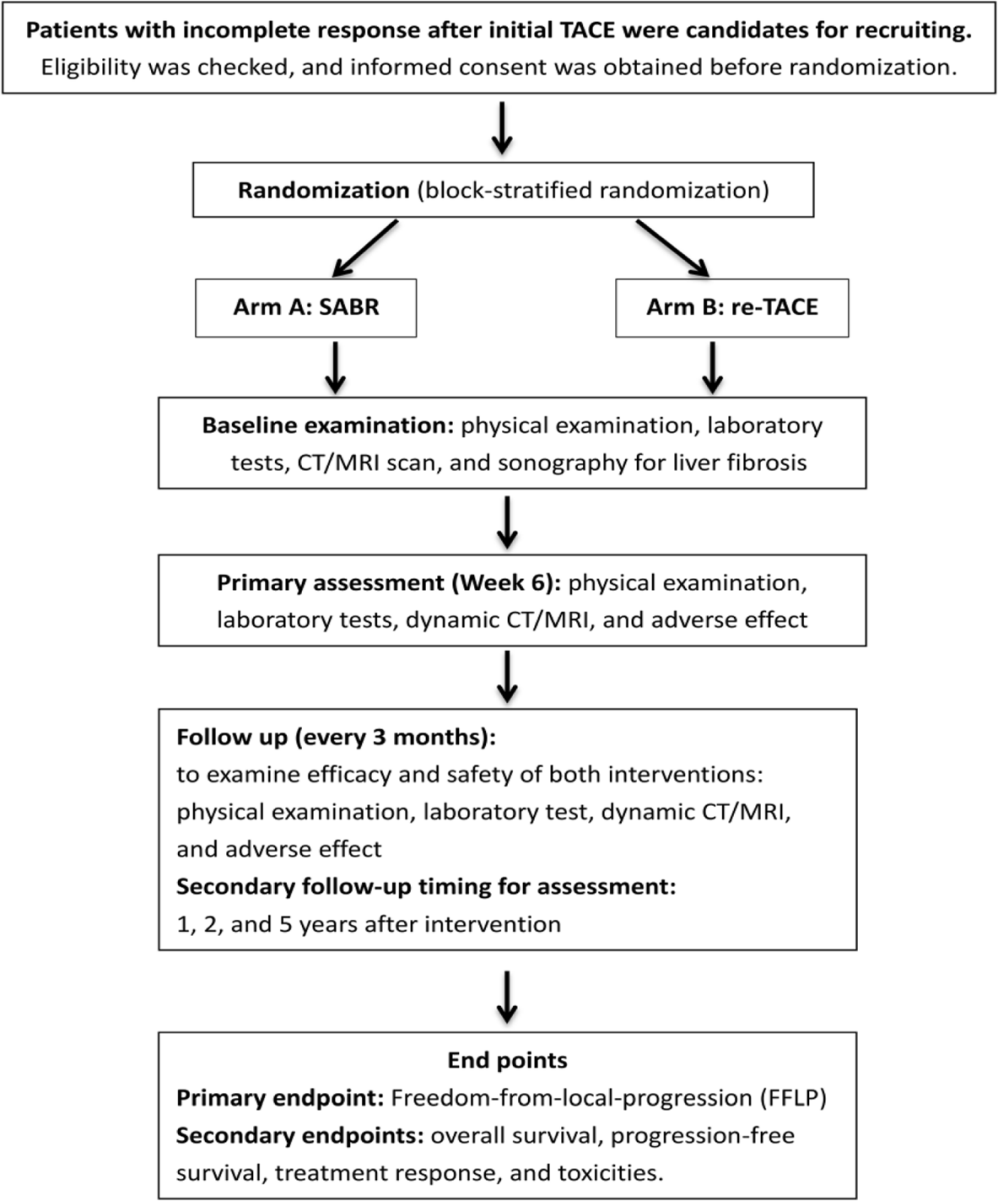
The study flow chart. Abbreviation: TACE, trans-arterial chemoembolization; SABR, stereotactic ablative radiotherapy; CT, computed tomography; MRI, magnetic resonance imaging. Note: The randomization will be conducted by using block-stratified method, intending to balance the patient number between groups

We will recruit patients who have incomplete response after their initial TACE. All recruited patients will be allocated randomly to the SABR or re-TACE group (Fig. 1).

### Arm A, the experimental group: SABR (planned *n* = 60)

SABR, or alternatively named Stereotactic Body Radiotherapy (SBRT), is a rapidly developing modern radiotherapy technique for liver tumors [47, 48]. TrueBeam™ (Varian Medical Systems, CA, USA) will be used to deliver SABR by using an extremely conformal radiation dose distribution and rapid fall-off of the peripheral dose, leading to a large sparing of surrounding normal tissues.

To achieve high precision, Image-guided Radiotherapy (IGRT) with respiratory-gated or breath-holding technique will be combined with SABR to overcome the effect of positioning uncertainty and respiratory motion. Real-time Position Management™ (RPM) system (Varian Medical System, CA, USA), version 1.7.5, will be used when breath-holding technique cannot be conducted confidentially. A CT scanner will be used for simulation (Somatom Emotion 6, Siemens Medical Solution, Forchheim, Germany). These techniques have been reported to improve treatment precision of irradiating targets that are affected by respiratory motion, such as lung, breast, and liver tumors [49].

SABR will be delivered in 5 fractions within 2 weeks, and the prescription dose will be ranged from 35 to 50 Gy, depending on tumor size, location, and normal tissue constrains. In some situations, treatment course will be prolonged to 8–10 fractions, for example, patients with poor liver function (e.g., Child B8–9) or their tumor location near the adjacent gastrointestinal tract, diaphragm, or the central zone of the liver (i.e., ≤1.5 cm around the main portal vein). The preferred inter-fraction time interval will be 48 h.

No expansion from Gross Target Volume (GTV) to CTV will be performed for most irradiating targets, and margins to generate Plan Target Volume (PTV) from Clinical Target Volume (CTV) will be ≤10 mm. The prescribed isodose should encompass 95% of PTV but 100% of CTV.

Critical organ constrains will be obeyed according to RTOG 1112 protocol [50]. Briefly, the 0.5-cc maximum dose regions of normal structures will be as follows: stomach, duodenum, small and large bowels, < 30 Gy; esophagus, < 32 Gy; and, 5 mm around the spinal cord, < 25 Gy. Bilateral mean kidney dose should be < 10 Gy. Allowed mean liver dose (MLD, the mean dose to the liver minus all GTV) should be kept < 13 Gy (if prescribed dose is 50 Gy) and < 15 Gy (if prescribed dose is 40–45 Gy).

Although the liver is considered as an organ with parallel functional subunits, the central hepatobiliary tract (cHBT) drained into the central hilum is more likely a serial structure. A 15-mm expansion of portal veins will be used as a surrogate for cHBT, and its constrains in 5-fraction SABR will be V26 < 37 c.c. and V21 < 45 c.c., as reported previously [51].

### Arm B, the active comparator group: re-TACE (planned *n* = 60)

TACE will be conducted by using a catheter to deliver chemotherapeutic agent and embolic materials into the blood vessels that supply to the tumor. Generally the procedure time will be completed within 1–2 h, and the hospitalization course will be 3 days.

Before each TACE, laboratory data will be checked, and abdominal images will be reviewed. Treatment plan will be discussed and determined by radiologists and gastrointestinal physicians. The planned catheter-inserted site will be the groin. A guide wire will be inserted into the femoral artery. A curve Angiographic Catheters (COOK MEDICAL INC., Bloomington, USA) followed by a superselective microcatheter (Terumo®, Tokyo, Japan) will be sent to the target artery in the liver.

As the catheter approach to the target, several series of angiography will be done to identify the lesion sites. After the target site is determined, catheter will be inserted to the feeding artery branch. Chemotherapy agent (Epirubicin, 30 mg), oil-based radio-opaque contrast agent (LIPIODOL® [ethiodized oil], 5–10 ml, according to tumor size), several 1 × 1 mm Sterile Sponge (Gelfoam®), contrast (XENETIX 350 [350 mg/ml], and solution for injection 10 ml) will be mixed together and then injected to the tumor site.

Post-procedure care will be prescribed, including keeping absolute bed rest for 12 h after the procedure. The incision wound will be compressed by a 2-Kg sandbag for 4–6 h for hemostasis. The medical care team will closely monitor vital signs, pulse of the distant limb, and awareness of whether TACE-associated complication occurred, such as nausea, vomiting, and allergic reactions. Proper treatments will be provided if complication occurs.

### Assignment of interventions: randomization and allocation

Computer-generated random numbers will be used for participant allocation. Two factors will be used as stratification factors, i.e., BCLC cancer stage and level of total bilirubin. A 2-by-2 cross table will be used for randomizing and blocking: total bilirubin (< 2 versus 2–3 g/dl) and BCLC (A versus B), with a block size of 4 in each stratification.

Opaque and sealed envelopes will be used for implementing the randomization and allocation sequence. When the participants are ready to be randomized, the study investigator will pull the very next randomization envelope by sequence from the envelope file, which will be locked in secure locker. The study nurse will record associated information on the study randomization list and the participant’s case report form. Finally, the envelope will be opened to reveal the subject’s treatment assignment and second study staff will double check the envelope file in real time to verify correct treatment assignment. An inspection of envelopes will be completed by data and safety monitoring board (DSMB) during routine monitoring visit. Note that blinding in either sides (i.e., physician or participants) cannot be done because of intervention natures.

### Criteria for discontinuing or modifying allocated interventions

All participants have full rights to request discontinuing intervention in any stage of trial allocation. For patients treated with SABR, if during-RT follow-up tests suggest impairment of liver function, such as GOT/GPT > 5-fold upper limit of normal range or total bilirubin > 2.0 g/dl, irradiation dose and treatment schedule will be considered to modify for consolidating patient safety.

### Strategies to improve adherence to intervention protocols

All participants will be followed up weekly during intervention period via both outpatient department (OPD) visit and phone contact, respectively. For patients with any grade 3 toxicity of CTCAE, in-patient care with aggressive management will be recommended and provided accordingly.

### Strategies for achieving adequate participant enrolment

Several strategies will be conducted for achieving an adequate enrolment of study participants. First, IRB-approved recruitment materials, including hard copy and electronic documents, will be posted in suitable platforms of hospitals and communities. Second, enrolment messages will be announced among national-level special societies, including – but not limited – gastrointestinal, surgical oncology, internal oncology, and radiation oncology. Third, inter-institute cooperation for potential patient enrolments may be done under formal supervision of IRBs.

### Relevant concomitant care and interventions that are permitted or prohibited during the trial

For all participants, relevant concomitant care and interventions for managing medical comorbidities, such as hypertension, type II diabetes mellitus, and hepatitis B/C infection, will be permitted. Permitted targeted therapy for HCC will be allowed to be prescribed subsequently – but not concomitantly – with SABR. However, chemotherapy agents, including intra-venous or oral forms, may be prohibited before a formal agreement of investigator committee.

## Follow-up measurement, assessment and outcomes

### Timing of assessments

Participants will be recruited from Dec. 2016 to Dec. 2019. All recruited participants will be followed up according to National Comprehensive Cancer Network (NCCN) guideline [6]. Table 1 shows a follow-up schedule and checklist according to recommendation of SPIRIT 2013 [46]. Follow-up data will be collected per 3 month since the baseline till 2 years. The timing to decide treatment response will be defined at the week 6 from the date of baseline. Other clinical outcome data regarding patient survival, response rate, and treatment toxicity, will be recorded systemically by schedule.

**Table 1:** Timeline schedule for participants: screen, intervention, and follow-up

	Screening	Baseline/Enrollment Visit 0 (V0)	Treatment visit 1 (V1)	Follow up visit 2 (V2, week 6)	Follow up visit 3 (V3, week 12)	Follow up visit 4 (V4, week 24)	Follow up visit 5 (V5, week 36)	Follow up visit 6 (V6, week 48)	Follow up visit 7 (V7, week 60)	Follow up visit 8 (V8, week 72)	Follow up visit 9 (V9, week 84)	Follow up visit 10 (V10, week 96)
Informed consent form		✓										
Randomization		✓										
Demographics	✓											
Medical history	✓											
Physical examination^a^	✓	✓	✓	✓	✓	✓	✓	✓	✓	✓	✓	✓
Vital signs	✓	✓	✓	✓	✓	✓	✓	✓	✓	✓	✓	✓
Performance status	✓	✓	✓	✓	✓	✓	✓	✓	✓	✓	✓	✓
Concomitant medications	✓	✓	✓	✓	✓	✓	✓	✓	✓	✓	✓	✓
Hemotology^b^	✓	✓	✓	✓	✓	✓	✓	✓	✓	✓	✓	✓
Serum biochemistry^c^	✓	✓	✓	✓	✓	✓	✓	✓	✓	✓	✓	✓
Hepatic disease examination^d^	✓	✓										
Abdominal CT or MRI	✓				✓		✓		✓		✓	
Whole-body CT scan						✓		✓		✓		✓
EKG (as indicated)	✓											
Fibrosis evaluation (Acoustic Radiation Force Impulse; ARFI)		✓										
Pregnancy test (urine)^e^		✓										
Adverse event evaluation			✓	✓	✓	✓	✓	✓	✓	✓	✓	✓

^a^Inclusion of physical examinations for abdominal condition, ascites, encephalopathy, body temperature, weight, height, pulse, and blood pressure

^b^Inclusion of CBC/DC, PLT, WBC classification, ANC, and PT/APTT

^c^Inclusion of AST, ALT, Albumin, alkaline phosphatase, LDH, total protein, total bilirubin, direct bilirubin, BUN, Cr, sodium ([NA^+^]), potassium ([K^+^]), AFP, GGT, INR

^d^Inclusion of HBsAg and Anti-HCV. Note that the following items are selective: HBeAg, HBeAb, HBcAg, HBV DNA, and HCV-RNA

^e^Women with childbearing potential only

Note 1: It was desirable to follow the study calendar as outlined, despite progression disease (PD). For per monthly tests, +/− 1 week was permitted, and for per 3, 6 and 12 monthly tests, +/− 2 weeks were permitted

Note 2: This table was modified from recommendation of SPIRIT 2013 [46]

Abbreviation: *CBC/DC* complete blood count/differential count, *PLT* platelet, *WBC* white blood cell, *ANC* absolute neutrophil count, *PT/APTT* prothrombin time/activated partial thromboplastin time, *AST* Aspartate Aminotransferase, *ALT* alanine aminotransferase, *LDH l*actate dehydrogenase, *BUN* blood urea nitrogen, *Cr* Creatinine, *AFP* alpha-fetoprotein, *GGT* γ-Glutamyltransferase, *INR* international normalized ratio, *HAV* hepatitis A virus, *HBsAg* hepatitis B surface antigen, *HCV* hepatitis C virus, *CT* computed tomography, *MRI* magnetic resonance imaging

### Plans to promote participant retention and complete follow-up

All investigators, including a specific study nurse, will promote participants retention and follow-up by using a team-work manner. A trial-specific information platform will be used. Follow-up data will be checked at least weekly. Loss of follow-up will be aggressively avoided by using multidisciplinary efforts.

## Outcome measures

### Primary outcome measure

The primary outcome measure is freedom from local progression (FFLP) [24]. FFLP will be defined as time with no in-field progressive disease. Response for target lesions will be defined according to the RECIST version 1.1 and modified RECIST (mRECIST) [52]. The target lesions should show intra-tumor arterial enhancement on contrast-enhanced CT or MRI and can be accurately measured in at least one dimension as 1 cm or more [53, 54].

Several tumor responses will be defined by two independent physicians (one hepatic-specific radiologist and one radiation oncologist): complete response (CR), total disappearance; partial response (PR), 30% decrease in single longest diameter (SLD) of contrast-enhanced part; stable disease (SD), neither partial response nor progressive disease met; and, progressive disease (PD), 20% increase of SLD or new lesion(s).

### Secondary outcome measure

Several secondary outcomes will be measured at 1 and 2 years, as follows: (1), overall survival; (2), progression-free survival; (3), tumor response rate; and (4), toxicity. Post-intervention complete or partial response will be defined as having a tumor response. Treatment-associated toxicity will be classified according to CTCAE, v.4.03 [55]. Toxicity within 3 months after initiation of interventions will be recorded as acute toxicity. And, toxicity that developed after 3 months will be considered as late toxicity. Liver-specific toxicity including classic and non-classic radiation-induced liver disease (RILD) will also be assessed. Degree of toxicities will be documented, such as ascites (i.e., absent, mild to moderate, and severe/refractory) and encephalopathy (i.e., absent, mild [I-II], and severe [III-IV]).

Several image and laboratory studies will be conducted for assessing treatment response and toxicities. First, CT, MRI, or abdominal sonography will be done per 3 months within the first two-year follow-up, then per 6 months thereafter for another 3 years. Second, specific laboratory studies will be followed up regularly, such as AST, ALT, and Bilirubin (both total and direct; Table 1). All adverse and potential harming events will be systemically collected and then analyzed in investigator meetings. Severe adverse events will be reported to IRB within 2 weeks as the IRB-monitoring guideline.

### Salvage management after SABR or re-TACE

For allocated patients who have incomplete response after SBRT or re-TACE, several re-salvage modalities will be evaluated as the following order: (1), surgical resection; (2), RFA; (3), TACE; (4), SABR; and, (5), conventional RT. Note that re-SABR or conventional RT may not be indicated for patients with post-SABR recurrence because of limitation of normal tissue tolerance.

### Data collection methods

Data will be collected primarily through the use of a paper-based case report forms (CRF), which will be completed by a specific study nurse and then confirmed by a physician. Subsequently, the data will be keyed in to the electronic research-incorporated database system (i.e., IROIP) that will be securely hosted at the Dalin Tzu Chi Hospital. Two independent investigators will be assigned to audit the process of data collection and correctness of the data. Baseline data will be collected routinely, including age, sex, ethnicity, education, economic, chronic hepatitis, prior treatments of HCC, and relevant medical comorbidities. All collected clinical data will be cross-linked with electronic laboratory data from the hospital HIS system.

### Sample size calculation

According to prior studies, response rates have been reported: 38% in patients treated with TACE alone [37] and 76.6% in those treated with TACE then salvage SABR [44]. As a result, an absolute increase of 30% in response rate will be considered as having a clinical significance. By using probability of type I error (α, 0.05; and, power [1 - β], 80%), 46 patients will be required in both the two study groups, resulting in a total study cases of 92. With an estimate of drop-out rate of 20%, we will enroll an additional 24 patients to counter potential attrition, reaching a final sample size of 120.

## Data management and access to data

All data collected in this study will be kept strictly confidential. All information from patients will be protected and stored in our data security system against unauthorized access. Only members in research can access to records, data, and samples. All information or data concerning the study will not be approached by any unauthorized third party.

## Data monitoring and auditing

During the whole study process, data accuracy and security will be monitored regularly by Data Safety Monitor Board (DSMB). All members of DSMB are independent from the sponsor and competing interests.

DSMB will perform planned interim analyses, and its members will decide whether the present trial should be terminated if stopping criteria are fitted. Stopping criteria include early identifications of significant benefits (i.e., between-group difference of absolute response rate > 30%) or harms (i.e., between-group difference of grade ≥ 3 toxicities > 20%). Final decision to stop the trial will require full agreement of all DSMB members.

Adverse events should be reported to the DSMB and IRB simultaneously, especially unexpected or severe adverse events. For every adverse event, potential avoidable etiologies will be checked systemically by both investigators and audit members of DSMB.

## Statistical methods and data analysis

Data will be analyzed by using SAS (version 9.2; SAS Institute, Inc., Cary, NC, USA) and SPSS (version 17, SPSS Inc., Chicago, IL, USA), accordingly. Demographic data will be examined differences between groups by using chi-square test (for categorical variables) and Wilcoxon rank sum test (for continuous variables).

Time-to-event outcomes, such as FFLP (time to in-field target-tumor progression), progression-free (time to local, regional, or distance progression) and overall survival (time to patient death from any cause) will be evaluated by using Kaplan-Meier method. Curve difference between groups will be assessed by using log-rank test. Multivariate analysis will be done by using Cox proportional hazard regression. Hazard ratio with 95% confidence interval will be provided in conjunction with *p* values to demarcate effective size. All statistical analysis will be performed by using a two-tailed approach. A *p* value of < 0.05 will be considered as having a statistical significance.

## Discussion

### SABR has been reported as an effective modality in all-stage HCC patients, but evidence from phase-III randomized trials is largely lacking

For patients with intermediate stage of BCLC classification, most patients received TACE as their local-regional therapies. However, TACE alone seldom achieves satisfactory complete response and demonstrates a dismal 5-year survival rate of < 20% [56]. Recently, modern high-dose SABR achieves 60–100% tumor response rate when combined with TACE [11 17–22]. Phase I and II trials also showed promising results of SABR on local control (1-year, 87%; 2-year, 94.6%) and survival rate (1-year, 55%; 2-year, 68.7%) [24, 25]. Thus, based on these results, SABR has been suggested as an effective modality for HCC patients with early-stage lesions [19, 24, 57, 58], unresectable tumors (i.e., ≤10 cm) [59], and post-TACE residual tumor [20, 44]. However, level 1 evidence from randomized trials for liver SABR is largely lacking.

A lack of level-III evidence is the main barrier for recommending SABR as a standard of care in most international treatment guidelines. As a result, conducting randomized trials to demarcate the role of SABR in HCC patients is warranted, especially in the Asia-Pacific region, where HBV- and HCV-related HCCs are predominated [2, 3].

### Conventional radiotherapy

Previously, the role of conventional external beam radiation therapy has been limited to palliative or salvage setting for large unresectable HCC, portal vein thrombosis, obstructive jaundice, and failure of prior TACE/RFA [6]. Nowadays, recent advances in irradiating technology lead the radiotherapy from palliation to cure in HCC management [47]. Local response rates range from 40 to 90%, and the median survival range from 10 to 25 months when treated with RT with or without TACE [60]. Remarkably, a recently published randomized trial showed that TACE plus conventional external beam radiation therapy can prolong progression-free survival at 12 and 24 weeks in locally advanced HCC patients when compares with sorafenib treatment [61]. Thus, in the present study, conventional RT will be allowed as one of alternative modalities for managing HCC patients who have incomplete response after SABR (the Arm A) or re-TACE (the Arm B) and for those patients who cannot (or do not) receive re-salvage treatment of TACE, SABR, or RFA.

### Current status

Study enrolment has been started since Dec. 2016. Participant recruitment is expected to be completed in Dec. 2020.

### Limitations of the study

The major limitation of the present study is small sample size. The relatively small sample size may increase the likelihood of a Type II error that skews the results.

## Abbreviations

AFP: Alpha-fetoprotein
AJCC: American Joint Committee on Cancer
BCLC: Barcelona Clinic Liver Cancer
CR: Complete response
CT: Computed tomography
CTCAE: Common terminology criteria of adverse events
FFLP: Freedom from local progression
HBV: Hepatitis B virus
HCC: Hepatocellular carcinoma
HCV: Hepatitis C virus
IGRT: Image-guided radiotherapy
IMRT: Intensity-modified radiotherapy
IRB: Institute Review Board
mRECIST: modified Response Evaluation Criteria in Solid Tumors
MRI: Magnetic Resonance Imaging
NCCN: National comprehensive cancer network
OPD: Out-patient department
PD: Progressive disease
PR: Partial response
PTV: Plan target volume
PVT: Portal vein thrombosis
RFA: Radiofrequency ablation
RILD: Radiation-induced liver disease
RPM: Real-time position management
RT: Radiotherapy
SABR: Stereotactic ablative radiotherapy
SBRT: Stereotactic body radiotherapy
SD: Stable disease
SIB: Simultaneously integrated boost
SIEB: Simultaneously integrated inner-escalated boost
SPIRIT: Standard Protocol Items: Recommendations for Interventional Trials
TACE: Trans-catheter arterial chemoembolization
VMAT: Volumetric-modulated arc radiotherapy

## References

[1] Global Burden of Disease Cancer C, Fitzmaurice C, Dicker D, Pain A, Hamavid H, Moradi-Lakeh M, MacIntyre MF, Allen C, Hansen G, Woodbrook R et al: The global burden of Cancer 2013. JAMA Oncol, 2015, 1(4):505–527. 10.1001/jamaoncol.2015.0735 PMC4500822 26181261

[2] Zhu RX, Seto WK, Lai CL, Yuen MF (2016). Epidemiology of hepatocellular carcinoma in the Asia-Pacific region. Gut and liver.

[3] Chang IC, Huang SF, Chen PJ, Chen CL, Chen CL, Wu CC, Tsai CC, Lee PH, Chen MF, Lee CM (2016). The hepatitis viral status in patients with hepatocellular carcinoma: a study of 3843 patients from Taiwan liver Cancer network. Medicine.

[4] Chiang CJ, Lo WC, Yang YW, You SL, Chen CJ, Lai MS. Incidence and survival of adult cancer patients in Taiwan, 2002–2012. *Journal of the Formosan Medical Association = Taiwan yi zhi*. 2016. 10.1016/j.jfma.2015.10.011 26786251

[5] Kudo M, Trevisani F, Abou-Alfa GK, Rimassa L (2016). Hepatocellular carcinoma: therapeutic guidelines and medical treatment. Liver Cancer.

[6] NCCN Clinical Practice Guidelines in Oncology: Hepatobiliary Cancers [http://www.nccn.org/professionals/physician_gls/f_guidelines.asp#site]. 10.6004/jnccn.2009.0027 PMC4461147 19406039

[7] McMasters KM, Vauthey JN (2011). Hepatocellular carcinoma: targeted therapy and multidisciplinary care.

[8] Cha C, Fong Y, Jarnagin WR, Blumgart LH, DeMatteo RP (2003). Predictors and patterns of recurrence after resection of hepatocellular carcinoma. J Am Coll Surg.

[9] Oliveri RS, Wetterslev J, Gluud C (2011). Transarterial (chemo)embolisation for unresectable hepatocellular carcinoma. The Cochrane database of systematic reviews.

[10] Llovet JM, Bruix J (2003). Systematic review of randomized trials for unresectable hepatocellular carcinoma: chemoembolization improves survival. Hepatology.

[11] Grieco A, Pompili M, Caminiti G, Miele L, Covino M, Alfei B, Rapaccini GL, Gasbarrini G (2005). Prognostic factors for survival in patients with early-intermediate hepatocellular carcinoma undergoing non-surgical therapy: comparison of Okuda, CLIP, and BCLC staging systems in a single Italian Centre. Gut.

[12] Matsuo Y, Yoshida K, Nishimura H, Ejima Y, Miyawaki D, Uezono H, Ishihara T, Mayahara H, Fukumoto T, Ku Y (2016). Efficacy of stereotactic body radiotherapy for hepatocellular carcinoma with portal vein tumor thrombosis/inferior vena cava tumor thrombosis: evaluation by comparison with conventional three-dimensional conformal radiotherapy. J Radiat Res.

[13] Yoon K, Kwak J, Cho B, Park JH, Yoon SM, Lee SW, Kim JH. Gated Volumetric-Modulated Arc Therapy vs. Tumor-Tracking CyberKnife Radiotherapy as Stereotactic Body Radiotherapy for Hepatocellular Carcinoma: A Dosimetric Comparison Study Focused on the Impact of Respiratory Motion Managements. PLoS One. 2016;11(11) e0166927. 10.1371/journal.pone.0166927 PMC5119818 27875568

[14] Su TS, Liang P, Liang J, Lu HZ, Jiang HY, Cheng T, Huang Y, Tang Y, Deng X (2017). Long-term survival analysis of stereotactic ablative radiotherapy versus liver resection for small hepatocellular carcinoma. Int J Radiat Oncol Biol Phys.

[15] Seo YS, Kim MS, Yoo HJ, Jang WI, Paik EK, Han CJ, Lee BH (2016). Radiofrequency ablation versus stereotactic body radiotherapy for small hepatocellular carcinoma: a Markov model-based analysis. Cancer Med.

[16] Kimura T, Aikata H, Takahashi S, Takahashi I, Nishibuchi I, Doi Y, Kenjo M, Murakami Y, Honda Y, Kakizawa H (2015). Stereotactic body radiotherapy for patients with small hepatocellular carcinoma ineligible for resection or ablation therapies. Hepatol Res.

[17] Jung J, Yoon SM, Han S, Shim JH, Kim KM, Lim YS, Lee HC, Kim SY, Park JH, Kim JH (2015). Alpha-fetoprotein normalization as a prognostic surrogate in small hepatocellular carcinoma after stereotactic body radiotherapy: a propensity score matching analysis. BMC Cancer.

[18] Takeda A, Sanuki N, Eriguchi T, Kobayashi T, Iwabutchi S, Matsunaga K, Mizuno T, Yashiro K, Nisimura S, Kunieda E (2014). Stereotactic ablative body radiotherapy for previously untreated solitary hepatocellular carcinoma. J Gastroenterol Hepatol.

[19] Sanuki N, Takeda A, Oku Y, Mizuno T, Aoki Y, Eriguchi T, Iwabuchi S, Kunieda E (2014). Stereotactic body radiotherapy for small hepatocellular carcinoma: a retrospective outcome analysis in 185 patients. Acta Oncol.

[20] Takeda A, Sanuki N, Tsurugai Y, Iwabuchi S, Matsunaga K, Ebinuma H, Imajo K, Aoki Y, Saito H, Kunieda E (2016). Phase 2 study of stereotactic body radiotherapy and optional transarterial chemoembolization for solitary hepatocellular carcinoma not amenable to resection and radiofrequency ablation. Cancer.

[21] Kim JW, Seong J, Lee IJ, Woo JY, Han KH (2016). Phase I dose escalation study of helical intensity-modulated radiotherapy-based stereotactic body radiotherapy for hepatocellular carcinoma. Oncotarget.

[22] Guarneri A, Franco P, Trino E, Campion D, Faletti R, Mirabella S, Gaia S, Ragona R, Diotallevi M, Saracco G (2016). Stereotactic ablative radiotherapy in the treatment of hepatocellular carcinoma >3 cm. Med Oncol.

[23] Lo CH, Yang JF, Liu MY, Jen YM, Lin CS, Chao HL, Huang WY (2017). Survival and prognostic factors for patients with advanced hepatocellular carcinoma after stereotactic ablative radiotherapy. PLoS One.

[24] Wahl DR, Stenmark MH, Tao Y, Pollom EL, Caoili EM, Lawrence TS, Schipper MJ, Feng M (2016). Outcomes after stereotactic body radiotherapy or radiofrequency ablation for hepatocellular carcinoma. J Clin Oncol.

[25] Palma DA, Haasbeek CJ, Rodrigues GB, Dahele M, Lock M, Yaremko B, Olson R, Liu M, Panarotto J, Griffioen GH (2012). Stereotactic ablative radiotherapy for comprehensive treatment of oligometastatic tumors (SABR-COMET): study protocol for a randomized phase II trial. BMC Cancer.

[26] Jacob R, Turley F, Redden DT, Saddekni S, Aal AK, Keene K, Yang E, Zarzour J, Bolus D, Smith JK (2015). Adjuvant stereotactic body radiotherapy following transarterial chemoembolization in patients with non-resectable hepatocellular carcinoma tumours of >/= 3 cm. HPB (Oxford).

[27] Paik EK, Kim MS, Jang WI, Seo YS, Cho CK, Yoo HJ, Han CJ, Park SC, Kim SB, Kim YH (2016). Benefits of stereotactic ablative radiotherapy combined with incomplete transcatheter arterial chemoembolization in hepatocellular carcinoma. Radiat Oncol.

[28] Lo CH, Huang WY, Lee MS, Lin KT, Lin TP, Chang PY, Fan CY, Jen YM (2014). Stereotactic ablative radiotherapy for unresectable hepatocellular carcinoma patients who failed or were unsuitable for transarterial chemoembolization. Eur J Gastroenterol Hepatol.

[29] Yang JF, Lo CH, Huang WY (2016). Is stereotactic body radiotherapy better than radiofrequency ablation for the treatment of hepatocellular carcinoma?. J Clin Oncol.

[30] De Bari B, Ozsahin M, Bize P, Boussaha T, Deplanque G, Wagner D, Bourhis J, Denys A (2016). Can stereotactic body radiotherapy really be considered the preferred treatment in large hepatocellular carcinoma?. J Clin Oncol.

[31] Kwon JH, Bae SH, Kim JY, Choi BO, Jang HS, Jang JW, Choi JY, Yoon SK, Chung KW (2010). Long-term effect of stereotactic body radiation therapy for primary hepatocellular carcinoma ineligible for local ablation therapy or surgical resection. Stereotactic radiotherapy for liver cancer. BMC Cancer.

[32] Meng M, Wang H, Zeng X, Zhao L, Yuan Z, Wang P, Hao X (2015). Stereotactic body radiation therapy: a novel treatment modality for inoperable hepatocellular carcinoma. Drug Discov Ther.

[33] Bujold A, Massey CA, Kim JJ, Brierley J, Cho C, Wong RK, Dinniwell RE, Kassam Z, Ringash J, Cummings B (2013). Sequential phase I and II trials of stereotactic body radiotherapy for locally advanced hepatocellular carcinoma. J Clin Oncol.

[34] Huertas A, Baumann AS, Saunier-Kubs F, Salleron J, Oldrini G, Croise-Laurent V, Barraud H, Ayav A, Bronowicki JP, Peiffert D (2015). Stereotactic body radiation therapy as an ablative treatment for inoperable hepatocellular carcinoma. Radiother Oncol.

[35] Jang WI, Kim MS, Bae SH, Cho CK, Yoo HJ, Seo YS, Kang JK, Kim SY, Lee DH, Han CJ (2013). High-dose stereotactic body radiotherapy correlates increased local control and overall survival in patients with inoperable hepatocellular carcinoma. Radiat Oncol.

[36] Teraoka Y, Kimura T, Aikata H, Daijo K, Osawa M, Honda F, Nakamura Y, Morio K, Morio R, Hatooka M, et al. Clinical outcomes of stereotactic body radiotherapy for elderly patients with hepatocellular carcinoma. Hepatol Res. 2017. 10.1111/hepr.12916 28544062

[37] Marelli L, Stigliano R, Triantos C, Senzolo M, Cholongitas E, Davies N, Tibballs J, Meyer T, Patch DW, Burroughs AK (2007). Transarterial therapy for hepatocellular carcinoma: which technique is more effective? A systematic review of cohort and randomized studies. Cardiovasc Intervent Radiol.

[38] Veltri A, Moretto P, Doriguzzi A, Pagano E, Carrara G, Gandini G (2006). Radiofrequency thermal ablation (RFA) after transarterial chemoembolization (TACE) as a combined therapy for unresectable non-early hepatocellular carcinoma (HCC). Eur Radiol.

[39] Guo W, He X, Li Z, Li Y (2015). Combination of Transarterial chemoembolization (TACE) and radiofrequency ablation (RFA) vs. surgical resection (SR) on survival outcome of early hepatocellular carcinoma: a meta-analysis. Hepatogastroenterology.

[40] Choi BO, Choi IB, Jang HS, Kang YN, Jang JS, Bae SH, Yoon SK, Chai GY, Kang KM (2008). Stereotactic body radiation therapy with or without transarterial chemoembolization for patients with primary hepatocellular carcinoma: preliminary analysis. BMC Cancer.

[41] Honda Y, Kimura T, Aikata H, Nakahara T, Naeshiro N, Tanaka M, Miyaki D, Nagaoki Y, Kawaoka T, Takaki S (2014). Pilot study of stereotactic body radiation therapy combined with transcatheter arterial chemoembolization for small hepatocellular carcinoma. Hepatogastroenterology.

[42] Honda Y, Kimura T, Aikata H, Kobayashi T, Fukuhara T, Masaki K, Nakahara T, Naeshiro N, Ono A, Miyaki D (2013). Stereotactic body radiation therapy combined with transcatheter arterial chemoembolization for small hepatocellular carcinoma. J Gastroenterol Hepatol.

[43] Janoray G, Mornex F (2015). follow-up after stereotactic body radiation therapy for liver tumours: a review of the literature and recommendations. Cancer Radiother.

[44] Kang JK, Kim MS, Cho CK, Yang KM, Yoo HJ, Kim JH, Bae SH, Jung da H, Kim KB, Lee DH (2012). Stereotactic body radiation therapy for inoperable hepatocellular carcinoma as a local salvage treatment after incomplete transarterial chemoembolization. Cancer.

[45] Seo YS, Kim MS, Yoo SY, Cho CK, Choi CW, Kim JH, Han CJ, Park SC, Lee BH, Kim YH (2010). Preliminary result of stereotactic body radiotherapy as a local salvage treatment for inoperable hepatocellular carcinoma. J Surg Oncol.

[46] Chan AW, Tetzlaff JM, Altman DG, Dickersin K, Moher D (2013). SPIRIT 2013: new guidance for content of clinical trial protocols. Lancet.

[47] Meyer J (2007). IMRT, IGRT, SBRT : advances in the treatment planning and delivery of radiotherapy. Basel.

[48] Kirichenko A, Gayou O, Parda D, Kudithipudi V, Tom K, Khan A, Abrams P, Szramowski M, Oliva J, Monga D (2016). Stereotactic body radiotherapy (SBRT) with or without surgery for primary and metastatic liver tumors. HPB (Oxford).

[49] Habermehl D, Naumann P, Bendl R, Oelfke U, Nill S, Debus J, Combs SE (2015). Evaluation of inter- and intrafractional motion of liver tumors using interstitial markers and implantable electromagnetic radiotransmitters in the context of image-guided radiotherapy (IGRT) - the ESMERALDA trial. Radiat Oncol.

[50] Radiation Therapy Oncology Group**:** Protocol RTOG 1112. Randomized phase III study of sorafenib versus stereotactic body radiation therapy followed by sorafenib in hepatocellular carcinoma [https://www.rtog.org/Portals/0/RTOG%20Broadcasts/Attachments/1112_master_w_update_5.7.13.pdf].

[51] Toesca DA, Osmundson EC, Eyben RV, Shaffer JL, Lu P, Koong AC, Chang DT. Central liver toxicity after SBRT: An expanded analysis and predictive nomogram. Radiother Oncol. 2016. 10.1016/j.radonc.2016.10.024 27865544

[52] Seyal AR, Gonzalez-Guindalini FD, Arslanoglu A, Harmath CB, Lewandowski RJ, Salem R, Yaghmai V (2015). Reproducibility of mRECIST in assessing response to transarterial radioembolization therapy in hepatocellular carcinoma. Hepatology.

[53] Eisenhauer EA, Therasse P, Bogaerts J, Schwartz LH, Sargent D, Ford R, Dancey J, Arbuck S, Gwyther S, Mooney M (2009). New response evaluation criteria in solid tumours: revised RECIST guideline (version 1.1). Eur J Cancer.

[54] Lencioni R, Llovet JM (2010). Modified RECIST (mRECIST) assessment for hepatocellular carcinoma. Semin Liver Dis.

[55] National Cancer Institute: Common Terminology Criteria for Adverse Events (CTCAE) [https://ctep.cancer.gov/protocolDevelopment/electronic_applications/ctc.htm].

[56] Zhang X, Wang K, Wang M, Yang G, Ye X, Wu M, Cheng S (2017). Transarterial chemoembolization (TACE) combined with sorafenib versus TACE for hepatocellular carcinoma with portal vein tumor thrombus: a systematic review and meta-analysis. Oncotarget.

[57] Yoon SM, Lim YS, Park MJ, Kim SY, Cho B, Shim JH, Kim KM, Lee HC, Chung YH, Lee YS (2013). Stereotactic body radiation therapy as an alternative treatment for small hepatocellular carcinoma. PLoS One.

[58] Shiozawa K, Watanabe M, Ikehara T, Matsukiyo Y, Kogame M, Kishimoto Y, Okubo Y, Makino H, Tsukamoto N, Igarashi Y (2015). Comparison of percutaneous radiofrequency ablation and CyberKnife((R)) for initial solitary hepatocellular carcinoma: a pilot study. World J Gastroenterol.

[59] Barry A, Knox JJ, Wei AC, Dawson LA (2016). Can stereotactic body radiotherapy effectively treat hepatocellular carcinoma?. J Clin Oncol.

[60] Hawkins MA, Dawson LA (2006). Radiation therapy for hepatocellular carcinoma: from palliation to cure. Cancer.

[61] Yoon SM, Ryoo BY, Lee SJ, Kim JH, Shin JH, An JH, Lee HC, Lim YS. Efficacy and safety of Transarterial chemoembolization plus external beam radiotherapy vs Sorafenib in hepatocellular carcinoma with macroscopic vascular invasion: a randomized clinical trial. JAMA Oncol. 2018. 10.1001/jamaoncol.2017.5847 PMC5885246 29543938

